# Measuring statistical evidence using relative belief

**DOI:** 10.1016/j.csbj.2015.12.001

**Published:** 2016-01-07

**Authors:** Michael Evans

**Affiliations:** Department of Statistics, University of Toronto

**Keywords:** Principle of empirical criticism, Checking for prior-data conflict, Statistical evidence, Relative belief ratios

## Abstract

A fundamental concern of a theory of statistical inference is how one should measure statistical evidence. Certainly the words “statistical evidence,” or perhaps just “evidence,” are much used in statistical contexts. It is fair to say, however, that the precise characterization of this concept is somewhat elusive. Our goal here is to provide a definition of how to measure statistical evidence for any particular statistical problem. Since evidence is what causes beliefs to change, it is proposed to measure evidence by the amount beliefs change from a priori to a posteriori. As such, our definition involves prior beliefs and this raises issues of subjectivity versus objectivity in statistical analyses. This is dealt with through a principle requiring the falsifiability of any ingredients to a statistical analysis. These concerns lead to checking for prior-data conflict and measuring the a priori bias in a prior.

## 1 Introduction

There is considerable controversy about what is a suitable theory of statistical inference. Given that statistical reasoning is used throughout science, it is important that such a theory be sound, in the sense that it is free from illogicalities and counterexamples, and be complete, in the sense that it produces unambiguous answers to all properly expressed statistical problems.

It is our contention that any such theory must deal explicitly with the concept of statistical evidence. Statistical evidence is much referred to in the literature, but most theories fail to address the topic by prescribing how it should be measured and how inferences should be based on this. The purpose of this paper is to provide an outline of a theory based on an explicit measure of statistical evidence.

Before describing this, there are several preliminary issues that need to be discussed. To start, we are explicit about what could be seen as the most basic problem in statistics and to which all others are related.

Example 1 The Archetypal Statistical Problem.

Suppose there is a population Ω with #(Ω) < ∞. So Ω is just a finite set of objects. Furthermore, suppose that there is a measurement *X* : *Ω* → *χ*. As such *X*(*ω*) ∈ *χ* is the measurement of object *ω* ∈ Ω.

This leads to the fundamental object of interest in a statistical problem, namely, the relative frequency distribution of *X* over Ω or, equivalently, the relative frequency function *f*_*X*_(*x*) = # ({*ω* : X(*ω*) = x})/ # (Ω) for x∈X. Notice that the frequency distribution is defined no matter what the set *χ* is. Typically, only a subset {*ω*_1_, … , *ω*_*n*_} ⊂ Ω can be observed giving the data *x*_i_ = *X*(*ω*_i_) for *i* = 1 , … , *n* where *n* ≪ # (Ω), so there is uncertainty about *f*_*X*_.

The standard approach to dealing with the uncertainty concerning *f*_*X*_ is to propose that *f*_*X*_ ∈ {*f*_*θ*_ : *θ* ∈ Θ}, a collection of possible distributions, and referred to as the statistical model. Due to the finiteness of Ω, and the specific accuracy with which *X*(*ω*) is measured, the parameter space Θ is also finite.

Note that in Example 1 there are no infinities and everything is defined simply in terms of counting.

So the position taken here is that in statistical problems there are essentially no infinities and there are no continuous distributions. Infinity and continuity are employed as simplifying approximations to a finite reality. This has a number of consequences, for example, any counterexample or paradox that depends intrinsically on infinity is not valid. Also, densities must be defined as limits as in *f*_*θ*_(*x*) = lim_ϵ → 0_*P*_*θ*_(*N*_ϵ_(*x*))/*Vol*(*N*_ϵ_(*x*)) where *N*_ϵ_(*x*) is a set that shrinks nicely to *x*, as described in Rudin [27], so *P*_*θ*_(*N*_ϵ_(*x*)) ≈ *f*_*θ*_(*x*)*Vol*(*N*_ϵ_(*x*)) for small ϵ.

To define a measure of evidence we need to add one more ingredient, namely, a prior probability distribution as represented by density π on Θ. For some, the addition of the prior will seem immediately objectionable as it is supposed to reflect beliefs about the true value of *θ* ∈ Θ and as such is subjective and so unscientific. Our answer to this is that all the ingredients to a statistical analysis are subjective with the exception, at least when it is collected correctly through random sampling, of the observed data. For example, a model {*f*_*θ*_ : *θ* ∈ Θ} is chosen and there is typically no greater foundation for this than it is believed to be reasonable, for example, this could be a set of normal distributions with unknown mean and variance.

The subjective nature of any statistical analysis is naturally of concern in scientific contexts as it is reasonable to worry about the possibility of these choices distorting what the data is saying through the introduction of bias. We cope with this, in part, through the following principle.

Principle of empirical criticism: Every ingredient chosen by a statistician as part of a statistical analysis must be checked against the observed data to determine whether or not it makes sense.

This supposes that the data, which hereafter is denoted by *x*, has been collected appropriately and so can be considered as being objective.

Model checking, where it is asked if the observed data is surprising for each *f*_*θ*_ in the model, is a familiar process and so the model satisfies this principle. It is less well-known that it is possible to provide a consistent check on the prior by assessing whether or not the true value of *θ* is a surprising value for *π*. Such a check is carried out by computing a tail probability based on the prior predictive distribution of a minimal sufficient statistic (see Evans and Moshonov [20], [21]). In Evans and Jang [16] it is proved that this tail probability is consistent in the sense that, as the amount of data grows, it converges to a probability that measures how far into the tails of the prior the true value of *θ* lies. Here “lying in the tails” is interpreted as indicating that a prior-data conflict exists since the data is not coming from a distribution where the prior assigns most of the belief. In Evans and Jang [17] it is shown how this approach to assessing prior-data conflict can be used to characterize weakly informative priors and also how to modify a prior, when such a conflict is obtained, in a way that is not data dependent, to avoid such a conflict. Further details and discussion on all of this can be found in Evans [13]. As such, the prior satisfies this principle as well. Just as with model checking, if the prior passes its checks this does not mean that the prior is correct, only that beliefs about *θ*, as presented by the prior, have not been contradicted by the data.

It is to be noted that, for any minimal sufficient statistic *T*, the joint probability measure Π × *P*_*θ*_ for (*θ*, *x*) factors as Π × *P*_*θ*_ = Π(⋅| *T*) × *M*_*T*_ × *P*(⋅| *T*) where *P*(⋅| *T*) is conditional probability of the data given *T*, *M*_*T*_ is the prior predictive for *T* and Π(⋅| *T*) is the posterior for *θ*. These probability measures are used respectively for model checking, checking the prior and for inference about *θ* and, as such, these activities are not confounded. Hereafter, it is assumed that the model and prior have passed their checks so we focus on inference. It is not at all clear that any other ingredients, such as loss functions, can satisfy the principle of empirical criticism but, to define a measure of evidence nothing beyond the model and the prior is required, so this is not a concern.

Given a model {*f*_*θ*_ : *θ* ∈ Θ}, a prior π and data *x*, we pose the basic problems of statistical inference as follows. There is a parameter of interest Ψ : Θ → Ψ (we do not distinguish between the function and its range to save notation) and there are two basic inferences.

Estimation: Provide an estimate of the true value of *ψ* = Ψ(*θ*) together with an assessment of the accuracy of the estimate.

Hypothesis assessment: Provide a statement of the evidence that the hypothesis *H*_0_ : Ψ(*θ*) = *ψ*_0_ is either true or false *together with an assessment of the strength of this evidence*.

Some of the statement concerning hypothesis assessment is in italics because typically the measure of the strength of the evidence is not separated from the statement of the evidence itself. For example, large values for Bayes factors and very small *p*-values are often cited as corresponding to strong evidence. In fact, separating the measure of evidence from a measure of its strength helps to resolve various difficulties.

There are of course many discussions in the statistical literature concerning the measurement of evidence. Chapter 3 of Evans [13] contains extensive analyses of many of these and documents why they cannot be considered as fully satisfactory treatments of statistical evidence. For example, sections of that text are devoted to discussions of pure likelihood theory, frequentist theory and *p*-values, Bayesian theories and Bayes factors, and fiducial inference. Some of the salient points are presented in the following paragraphs together with further references.

Edwards [10] and Royall [26] develop an approach to inference based upon recognizing the centrality of the concept of statistical evidence and measuring this using likelihood ratios for the full model parameter *θ*. A likelihood ratio, however, is a measure of relative evidence between two values of *θ* and is not a measure of the evidence that a particular value *θ* is true. The relative belief ratio for *θ*, defined in Section 2, is a measure of the evidence that *θ* is true and furthermore a calibration of this measure of evidence is provided. While these are significant differences in the two approaches, there are also similarities between the pure likelihood approach and relative belief approach to evidence. For example, it is easily seen that the relative belief ratio for *θ* gives the same ratios between two values as the likelihood function. Another key difference arises, however, when considering measuring evidence for an arbitrary *ψ* = Ψ(*θ*). Pure likelihood theory does not deal with such marginal parameters in a satisfactory way and the standard recommendation is to use a profile likelihood. A profile likelihood is generally not a likelihood and so the basic motivating idea is lost. By contrast the relative belief ratio for such a *ψ* is defined in a consistent way as a measure of change in belief.

In frequency theory *p*-values are commonly used as measures of evidence. A basic issue that arises with the *p*-value is that a large value of such a quantity cannot be viewed as evidence that a hypothesis is true. This is because in many examples, a *p*-value is uniformly distributed when the hypothesis is true. It seems clear that any valid measure of evidence must be able to provide evidence for something being true as well as evidence against and this is the case for the relative belief ratio. Another key problem for *p*-values arises with so-called “data snooping” as discussed in Cornfield [6] where an investigator who wants to use the standard 5% value for significance can be prevented from ever attaining significance if they obtain a slightly larger value for a given sample size and then want to sample further to settle the issue. Royall [26] contains a discussion of many of the problems associated with *p*-values as measures of evidence. A much bigger issue for a frequency theory of evidence is concerned with the concept of ancillary statistics and the conditionality principle. The lack of a unique maximal ancillary leads to ambiguities in the characterization of evidence as exemplified by the discussion in Birnbaum [2], Evans, Fraser and Monette [14] and Evans [12]. A satisfactory frequentist theory of evidence requires a full resolution of this issue. The book Taper and Lele [29] contains a number of papers discussing the concept of evidence in the frequentist and pure likelihood contexts.

In a Bayesian formulation the Bayes factor is commonly used as a measure of evidence. The relationship between the Bayes factor and the relative belief ratio is discussed in Section 2. It is also the case, however, that posterior probabilities are used as measures of evidence. Relative belief theory, however, draws a sharp distinction between measuring beliefs, which is the role of probability, and measuring evidence, which is measured by change in beliefs from a priori to a posteriori. As discussed in the following sections, being careful about this distinction is seen to resolve a number of anomalies for inference. Closely related to Bayesian inference is entropic inference as discussed, for example, in Caticha [3], [4]. In entropic inference relative entropy plays a key role in determining how beliefs are to be updated after obtaining information. This is not directly related to relative belief as discussed here, although updating beliefs via conditional probability is central to the approach and so there are some points in common. Another approach to measuring statistical evidence, based on a thermodynamical analogy, can be found in Vieland [31].

The Dempster–Shafer theory of belief functions, as presented in Shafer [28], is another approach to the development of a theory of evidence. This arises by extending the usual formulation of probability, as the measure of belief in the truth of a proposition, to what could be considered as upper and lower bounds on this belief. While this clearly distinguishes the theory of belief functions from relative belief, a more fundamental distinction arises from measuring evidence via a change in belief in the relative belief approach as opposed to using probability itself or bounds based on probabilities. Cuzzolin [8] discusses a mathematical function mapping a belief function to a probability measure called the relative belief transform. Basically the relative belief transform of a belief function defined on a finite set, is the probability function obtained by normalizing the belief function restricted to singleton sets. As will be seen in Section 2, this is not related to the relative belief ratio as a measure of evidence.

## 2 The relative belief ratio and inferences

To determine inferences three simple principles are needed. First is the principle of conditional probability that tells us how beliefs should change after receiving evidence bearing on the truth of an event. We let Ω denote a general sample space for response *ω* with associated probability measure *P*.

The principle of conditional probability: For events *A* , *C* ⊂ Ω with *P*(*C*) > 0, if told that the event *C* has occurred, then replace *P*(*A*) by $P\left(A\;|\;C\right)=P\left(A\cap C\right)/P\left(C\right).$

This leads to a very simple characterization of evidence.

Principle of evidence: If *P*(*A* | *C*) > *P*(*A*), then there is evidence in favor of *A* being true because the belief in *A* has increased. If *P*(*A* | *C*) < *P*(*A*), then there is evidence *A* is false because the belief in *A* has decreased. If *P*(*A* | *C*) = *P*(*A*), then there isn't evidence either in favor of *A* or against *A* as belief in *A* has not changed.

This principle suggests that any valid measure of the quantity of evidence is a function of (*P*(*A*), *P*(*A* | *C*)). A number of such measures have been discussed in the literature and Crupi et al. [7] contains a nice survey. A detailed examination in Evans [13] leads to selecting the relative belief ratio as the most natural as virtually all the others are either equivalent to this or do not behave properly in the limit for continuous models.

Principle of relative belief: The evidence that *A* is true, having observed *C*, is measured by the relative belief ratio *RB*(*A* | *C*) = *P*(*A* | *C*)/*P*(*A*) when *P*(*A*) > 0.

So, for example, *RB*(*A* | *C*) > 1 implies that observing *C* is evidence in favor of *A* and the bigger *RB*(*A* | *C*) is, the more evidence in favor.

The Bayes factor is also used as a measure of evidence. The Bayes factor *BF*(*A* | *C*) in favor of *A* being true is the ratio of the posterior to prior odds in favor of *A*. It is easily shown that *BF*(*A* | *C*) = *RB*(*A* | *C*)/*B*(*A*^*c*^ | *C*), namely, from the point of view of the relative belief ratio, the Bayes factor is a comparison between the evidence in favor of *A* and the evidence in favor of its negation. The relative belief ratio satisfies *RB*(*A* | *C*) = *BF*(*A* | *C*)/(1 − *P*(*A*) + *P*(*A*)*BF*(*A* | *C*)) and so cannot be expressed in terms of the Bayes factor itself. From this it is concluded that the relative belief ratio is a somewhat more elemental measure of evidence. As discussed in Baskurt and Evans [1] and Evans [13], the relative belief ratio is preferred as a measure of evidence as it leads to a much simpler theory of inference.

For the statistical context suppose interest is in *ψ* = Ψ(*θ*). Let *π*_Ψ_(⋅| *x*) and *π*_Ψ_ denote the posterior and prior densities of *ψ*. Then the three principles imply that the relative belief ratio

$$RB_{\Psi }\left(\psi \;|\;x\right)=\pi_{\Psi }\left(\psi \;|\;x\right)/\pi_{\Psi }\left(\psi \right)$$

is the appropriate measure of the evidence that *ψ* is the true value and this holds as a limit in the continuous case, see Evans [13]. Also, in the continuous case, the limiting value of the Bayes factor is given by *RB*_Ψ_(*ψ* | *x*) so the measures agree in that context. Given *RB*_Ψ_(⋅| *x*), this prescribes a total order for the *ψ* values as *ψ*_1_ is not preferred to *ψ*_2_ whenever *RB*_Ψ_(*ψ*_1_ | *x*) ≤ *RB*_Ψ_(*ψ*_2_ | *x*) since there is at least as much evidence for *ψ*_2_ as there is for *ψ*_1_. This in turn leads to unambiguous solutions to the inference problems.

### 2.1 Estimation

The best estimate of *ψ* is the value for which the evidence is greatest, namely,

$$\psi \left(x\right)=\arg\sup RB_{\Psi }\left(\psi |x\right),$$

and called the least relative surprise estimator in Evans [11], Evans and Shakhatreh [22] and Evans and Jang [18]. Associated with this is a γ-relative belief credible region

$$C_{\Psi ,\gamma }\left(x\right)=\left\{\psi :RB_{\Psi }\left(\psi |x\right)\geq c_{\Psi ,\gamma }\left(x\right)\right\}$$

where *c*_Ψ , γ_(*x*) = inf {*k* : Π_Ψ_(*RB*_Ψ_(*ψ* | *x*) ≤ *k* | *x*) ≥ 1 − *γ*}. Notice that *ψ*(*x*) ∈ *C*_Ψ , *γ*_(*x*) for every γ ∈ [0, 1] and so, for selected *γ*, the size of *C*_Ψ , γ_(*x*) can be taken as a measure of the accuracy of the estimate *ψ*(*x*). Given the interpretation of *RB*_Ψ_(*ψ* | *x*) as the evidence for *ψ*, we are forced to use the sets *C*_Ψ , γ_(*x*) for the credible regions. For if *ψ*_1_ is in such a region and *RB*_Ψ_(*ψ*_2_ | *x*) ≥ *RB*_Ψ_(*ψ*_1_ | *x*), then *ψ*_2_ must be in the region as well as there is at least as much evidence for *ψ*_2_ as for *ψ*_1_. This presents the relative belief solution to the Estimation problem.

### 2.2 Hypothesis assessment

For the assessment of the hypothesis *H*_0_ : Ψ(*θ*) = *ψ*_0_, the evidence is given by *RB*_Ψ_(*ψ*_0_ | *x*). One problem that both the relative belief ratio and the Bayes factor share as measures of evidence, is that it is not clear how they should be calibrated. Certainly the bigger *RB*_Ψ_(*ψ*_0_ | *x*) is than 1, the more evidence there is in favor of *ψ*_0_ while the smaller *RB*_Ψ_(*ψ*_0_ | *x*) is than 1, the more evidence there is against *ψ*_0_. But what exactly does a value of *RB*_Ψ_(*ψ*_0_ | *x*) = 20 mean? It would appear to be strong evidence in favor of *ψ*_0_ because beliefs have increased by a factor of 20 after seeing the data. But what if other values of *ψ* have even larger increases?

The value *RB*_Ψ_(*ψ*_0_ | *x*) can be calibrated, however, by comparing it to the other possible values *RB*_Ψ_(⋅| *x*) through its posterior distribution. For example, one possible measure of the strength is

(1) $$\Pi_{\Psi }\left(RB_{\Psi }\left(\psi |x\right)\leq RB_{\Psi }\left(\psi_{0}|x\right)|x\right)$$

which is the posterior probability that the true value of *ψ* has a relative belief ratio no greater than that of the hypothesized value *ψ*_0_. While Eq. (1) may look like a *p*-value, it has a very different interpretation. For when *RB*_Ψ_(*ψ*_0_ | *x*) < 1, so there is evidence against *ψ*_0_, then a small value for Eq. (1) indicates a large posterior probability that the true value has a relative belief ratio greater than *RB*_Ψ_(*ψ*_0_ | *x*) and there is strong evidence against *ψ*_0_. If *RB*_Ψ_(*ψ*_0_ | *x*) > 1, so there is evidence in favor of *ψ*_0_, then a large value for Eq. (1) indicates a small posterior probability that the true value has a relative belief ratio greater than *RB*_Ψ_(*ψ*_0_ | *x*) and so there is strong evidence in favor of *ψ*_0_. Notice that, in the set {*ψ* : *RB*_Ψ_(*ψ* | *x*) ≤ *RB*_Ψ_(*ψ*_0_ | *x*)}, the “best” estimate of the true value is given by *ψ*_0_ simply because the evidence for this value is the largest in this set.

Various results have been established in Baskurt and Evans [1] supporting both *RB*_Ψ_(*ψ*_0_ | *x*), as the measure of the evidence, and Eq. (1), as a measure of the strength of that evidence. For example, the following simple inequalities are useful in assessing the strength, namely:

$$\begin{array}{l} \Pi_{\Psi }\left(RB_{\Psi }\left(\psi |x\right)=RB_{\Psi }\left(\psi_{0}|x\right)|x\right)\leq \Pi_{\Psi }\left(RB_{\Psi }\left(\psi |x\right)\leq RB_{\Psi }\left(\psi_{0}|x\right)|x\right)\\ \;\leq RB_{\Psi }\left(\psi_{0}|x\right). \end{array}$$

So if *RB*_Ψ_(*ψ*_0_ | *x*) > 1 and Π_Ψ_({*RB*_Ψ_(*ψ*_0_ | *x*)}| *x*) is large, there is strong evidence in favor of *ψ*_0_ while, if *RB*_Ψ_(*ψ*_0_ | *x*) < 1 is very small, then there is immediately strong evidence against *ψ*_0_.

To see more clearly the issue concerning calibration consider the following basic example. Suppose that the data *x* is a sample of *n* from a *N*(*μ*, *σ*^2^) distribution, with *μ* ∈ *R*^1^ unknown and σ^2^ known, and the prior is given by a *N*(*μ*_0_, *τ*_0_^2^) distribution. It is common to take *τ*_0_^2^ very large to reflect the lack of much prior information about the true value of *μ*. But it is easily shown that (see Baskurt and Evans [1] or Evans [13]), for any particular value of *μ*, then *RB*(*μ* | *x*) → ∞ as *τ*_0_^2^ → ∞ and this is also true of the Bayes factor as it equals *RB*(*μ* | *x*) in this case. So by being appropriately uninformative about the true value of *μ*, one can make the evidence in favor of a particular value of *μ* as large as one likes. This example also produces the Jeffreys–Lindley paradox because it is possible that the classical frequentist *p*-value is very small when assessing the hypothesis that *μ*_0_ is the true value, while the corresponding relative belief ratio/Bayes factor is large in favor of this hypothesis and so these measures contradict each other. When the relative belief ratio is calibrated, however, the classical *p*-value is seen to arise as a measure of the strength of the evidence and so this says that, while there may be evidence in favor of *μ*_0_, it may be weak evidence. It is clear that by choosing the prior to be very diffuse a bias in favor of the hypothesis is being introduced and the final resolution of the paradox is accomplished by computing what is referred to as bias in favor, as is discussed in the following section. This example makes it clear that the value of a relative belief ratio or Bayes factor cannot be interpreted generally as a measure of the strength of the evidence.

### 2.3 Bias

There is another issue associated with using *RB*_Ψ_(*ψ*_0_ | *x*) to assess the evidence that *ψ*_0_ is the true value. One of the key concerns with Bayesian inference methods is that the choice of the prior can bias the analysis in various ways. An approach to dealing with the bias issue is discussed in Baskurt and Evans [1]. Given that the assessment of the evidence that *ψ*_0_ is true is based on *RB*_Ψ_(*ψ*_0_ | *x*), the solution is to measure a priori whether or not the chosen prior induces bias either in favor of or against *ψ*_0_. To see how to do this, note first the Savage–Dickey ratio result (see Dickey [9]), which says that

(2) $$RB_{\Psi }\left(\psi_{0}|x\right)=m\left(x|\psi_{0}\right)/m\left(x\right)$$

where $m\left(x|\psi_{0}\right)=\int_{\left\{\theta :\Psi \left(\theta \right)=\psi_{0}\right\}}\pi \left(\theta |\psi_{0}\right)f_{\theta }\left(x\right)\;\mathrm{d\theta}$ is the conditional prior-predictive density of the data *x* given that Ψ(*θ*) = *ψ*_0_ and $m\left(x\right)=\int_{\Theta }\mu \left(\theta \right)f_{\theta }\left(x\right)\;\mathrm{d\theta}$ is the prior-predictive density of the data *x*.

From Eq. (2) the bias in the evidence against *ψ*_0_ can be measured by computing

(3) $$M\left(\left(m\left(x|\psi_{0}\right)/m\left(x\right)\leq 1\;\right|\psi_{0}\right),$$

where *M*(⋅|* ψ*_0_) is the prior probability measure of the data given that *ψ*_0_ is the true value. Therefore, Eq. (3) is the prior probability that evidence for *ψ*_0_ will not be obtained when *ψ*_0_ is true. So when Eq. (3) is large there is bias against *ψ*_0_ and subsequently reporting that there is evidence against *ψ*_0_ is not convincing. To measure the bias in favor of *ψ*_0_, choose values *ψ*_0_^'^ ≠ *ψ*_0_ such that the difference between *ψ*_0_ and *ψ*_0_^'^ represents the smallest difference of practical importance. Then compute

(4) $$M\left(\left(m\left(x|\psi_{0}\right)/m\left(x\right)\geq 1\right|\psi_{0}^{'}\right),$$

as this is the prior probability that evidence against *ψ*_0_ will not be obtained when *ψ*_0_ is false. Note that Eq. (4) tends to decrease as *ψ*_0_^'^ moves away from *ψ*_0_. When Eq. (4) is large, there is bias in favor of *ψ*_0_ and so subsequently reporting that evidence in favor of *ψ*_0_ being true has been found, is not convincing. For a fixed prior, both Eqs. (3) and (4) decrease with sample size and so, in design situations, they can be used to set sample size and so control bias (see Evans [13]). Considering the bias in the evidence is connected with the idea of a severe test as discussed in Popper [25] and Mayo and Spanos [23].

## 3 Examples

Consider now examples of applying relative belief inferences. The first example is concerned with making inferences about an unknown proportion.

Example 2 Inferences for a proportion.

Suppose that *x* = (*x*_1_, … , *x*_*n*_) ∈ {0, 1}^*n*^ is observed where the *x*_*i*_ are assumed to be i.i.d. Bernoulli(*θ*) with *θ* ∈ [0, 1]. This could arise from tossing a coin *n* times where 1 denotes a head and 0 a tail and *θ* is the probability of obtaining a head. A beta(*α*_0_, *β*_0_) distribution, where *α*_0_ and *β*_0_ are specified, is taken for the prior. Let the parameter of interest be Ψ(*θ*) = *θ*. The the posterior of *θ* is a beta$\left(n\overset{̅}{x}+\alpha_{0},n-n\overset{̅}{x}+\beta_{0}\right)$ distribution. Let us suppose for this example that, based on an elicitation, it is believed *α*_0_ = *β*_0_ = 4 provides an appropriate prior so the posterior is a beta$\left(n\overset{̅}{x}+4,n-n\overset{̅}{x}+4\right)$ distribution.

Suppose the data is given by

(5) $$x=\left(1,1,0,0,0,0,0,0,0,0,1,1,1,1,0,1,0,0,1,0\right).$$

**Fig. 1:**
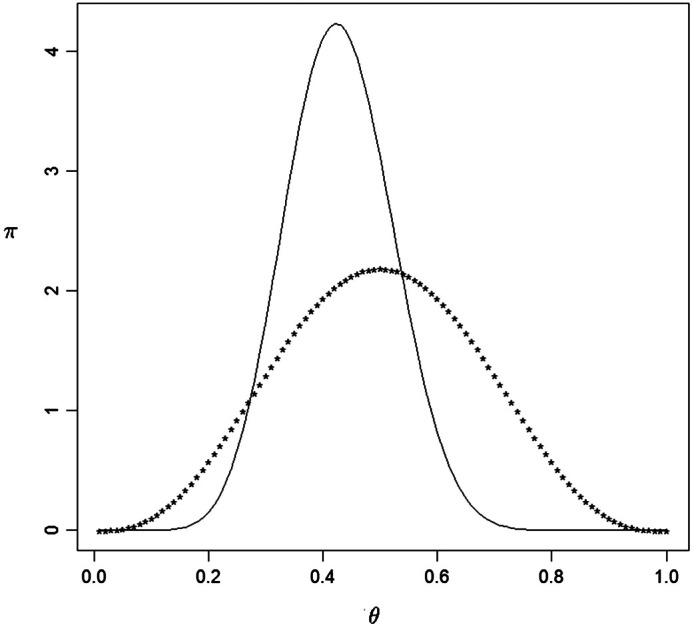
The prior *** and the posterior — densities in Example 2.

This data was actually generated from a Bernoulli(1/2) so indeed procedures for model checking and checking for prior-data conflict do not find any issues with the choices made. Fig. 1 is a plot of the beta(4, 4) prior together with the beta(12, 16) posterior based on this data. Clearly the data has lead to some learning concerning the true value of *θ*.

**Fig. 2:**
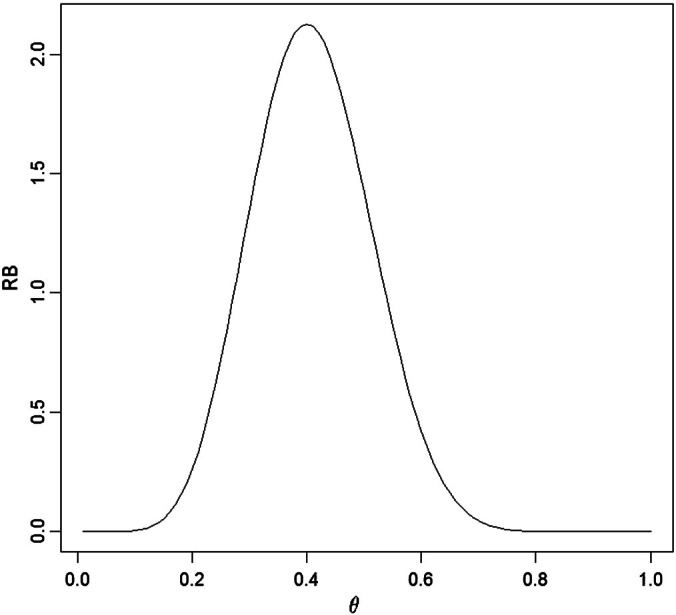
Plot of *RB*(*θ* | *x*) in Example 2.

For this situation

$$\mathrm{RB}\left(\theta \;|\;x\right)=\frac{\pi \left(\theta \;|\;n\overset{̅}{x}\right)}{\pi \left(\theta \right)}=\frac{\Gamma \left(\alpha_{0}\right)\Gamma \left(\beta_{0}\right)}{\Gamma \left(\alpha_{0}+\beta_{0}\right)}\frac{\Gamma \left(n+\alpha_{0}+\beta_{0}\right)}{\Gamma \left(n\overset{̅}{x}+\alpha_{0}\right)\Gamma \left(n-n\overset{̅}{x}+\beta_{0}\right)}\theta^{n\overset{̅}{x}}{\left(1-\theta \right)}^{n-n\overset{̅}{x}}$$

and this is plotted in Fig. 2.

When making inference about the full model parameter *θ* we always have *θ*(*x*) = *θ*_*MLE*_(*x*) which in this case is $\overset{̅}{x}=0.400$. To assess the accuracy of this estimate, we compute the 0.95-credible region

$$C_{0.95}\left(x\right)=\left\{\theta :\mathrm{RB}\left(\theta \;|\;x\right)\geq c_{0.95}\left(x\right)\right\}.$$

which is also a likelihood interval for *θ*. Here *C*_0.95_(*x*) = (0.227, 0.593) and its length 0.593 − 0.227 = 0.366 indicates that there is a reasonable degree of uncertainty about the true value of *θ*. Note that, while relative belief inferences for *θ* take the same form as likelihood inferences for *θ*, it is not correct to consider *RB*(⋅| *x*) as a likelihood function as multiplying it by a positive constant destroys its interpretation as a measure of evidence. For a general Ψ(*θ*), the relative belief ratio *RB*_Ψ_(⋅| *x*) is not proportional to a profile likelihood function.

To assess the hypothesis *H*_0_ : *θ* = *θ*_0_ compute *RB*(*θ*_0_ | *x*). In this case, when θ_0_ = 1/2, then *RB*(1/2 | *x*) = 1.421, and since this is greater than 1, there is evidence in favor of *H*_0_. For the strength of this evidence we obtain,

$$\Pi \left(\mathrm{RB}\left(\theta \;|\;x\right)\leq \mathrm{RB}\left(1/2\;|\;x\right)\;|\;n\overset{̅}{x}=8\right)=0.309$$

and conclude that the evidence in favor of *H*_0_ is only moderate as there is a posterior probability of 0.691 that the true value of *θ* has a larger relative belief ratio. It is wrong, however, to conclude from the value 0.691 that there is evidence against *θ*_0_ = 1/2 because indeed the data have lead to an increase in belief that this is the true value. At the same time it is reasonable to have some concern about the reliability of this inference since the strength is not large. To see what the strength represents graphically consider Fig. 2 and draw a horizontal line at height 0.309. This line intersects the graph of *RB*(⋅| *x*) at two points which, when projected onto the *θ*-axis, gives an interval of *θ* values. The strength is then the posterior content of the two tails that form the complement of this interval together with the end-points. This geometric interpretation generalizes in an obvious way to the situation where *θ* is multidimensional.

To assess the bias against *H*_0_ : *θ* = 1/2, compute the prior probability, when *H*_0_ is true, that evidence against *H*_0_ will be obtained, namely,

$$M\left(\frac{m\left(x\;|\;1/2\right)}{m\left(x\right)}\leq 1\;|\;\theta_{0}\right)=0.265.$$

This indicates only modest bias against *θ*_0_. Bias in favor of *H*_0_ : *θ* = 1/2 is measured by the prior probability, when *θ* = *θ*_⁎_ ∈ {0.45, 0.55} is true, that there is evidence in favor of *H*_0_, namely,

$$M\left(\frac{m\left(x\;|\;\theta_{0}\right)}{m\left(x\right)}>1\;|\;0.45\right)=0.692,M\left(\frac{m\left(x\;|\;\theta_{0}\right)}{m\left(x\right)}>1\;|\;0.55\right)=0.692.$$

So there is some bias in favor of *H*_0_ = {1/2} induced by the beta(4, 4) prior, at least when a deviation of 0.05 from the null is considered as meaningful. A smaller deviation considered as meaningful would result in more bias in favor of *H*_0_. As previously mentioned, both biases can be controlled, namely, made as small as desired, by choosing the sample size *n* appropriately.

The following example is very simple but nevertheless it has produced considerable confusion concerning the role of measuring evidence as opposed to taking a decision-theoretic approach to statistical inference. It emphasizes the importance of being very clear about how to measure evidence.

Example 3 Prosecutor's fallacy.

In general, the prosecutor's fallacy refers to any kind of error in probabilistic reasoning made by a prosecutor when arguing for the conviction of a defendant. The paper Thompson and Schumann [30] seems to be one of the earliest references and so that context and its relevance to measuring statistical evidence is considered.

Suppose a population is split into two classes where a proportion ϵ are guilty of a crime and a proportion 1 − ϵ are not guilty. Suppose further that a particular trait is held by a proportion ψ_1_ of those innocent and a proportion ψ_2_ of those who are guilty. The overall proportion in the population possessing the trait is then (1 − ϵ)ψ_1_ + ϵψ_2_ and this will be small whenever ϵ and ψ_1_ are small. The values ϵ and ψ_1_ being small correspond to the proportion of guilty being very small and the trait being very rare in the population. The prosecutor notes that the defendant has this trait and, because (1 − ϵ)ψ_1_ + ϵψ_2_ is very small, concludes the defendant is guilty. Actually, as cited in Thompson and Schumann [30], it seems that the prosecutor in question actually quoted 1 − {(1 − ϵ)ψ_1_ + ϵψ_2_} as the probability of guilt! In any case, our concern here is the fallacious reasoning concerning the smallness of (1 − ϵ)ψ_1_ + ϵψ_2_ and what it implies about the guilt of the defendant.

Treating ϵ as the prior probability that the defendant is guilty, without observing whether or not they have the trait, it is seen immediately that the posterior probability that the defendant is guilty, given that they have the trait, is

$$P\left(“\text{guilty}”|“\text{defendant}\;\mathrm{has}\;\text{the}\;\text{trait}”\right)=\frac{\epsilon \psi_{2}}{\left(1-\epsilon \right)\psi_{1}+\epsilon \psi_{2}}$$

and this converges to 0 as ϵ → 0. The relative belief ratio for guilt is

$$\mathrm{RB}\left(“\text{guilty}”|“\text{defendant}\;\mathrm{has}\;\text{the}\;\text{trait}”\right)=\frac{\psi_{2}}{\left(1-\epsilon \right)\psi_{1}+\epsilon \psi_{2}}$$

and the relative belief ratio for innocence is

$$\mathrm{RB}\left(“\text{innocent}”|“\text{defendant}\;\mathrm{has}\;\text{the}\;\text{trait}”\right)=\frac{\psi_{1}}{\left(1-\epsilon \right)\psi_{1}+\epsilon \psi_{2}}.$$

Now *RB* (“guilty” | “defendant has the trait”) > 1 if and only if ψ_2_ > ψ_1_ and this occurs if and only if *RB* (“innocent” |“defendant has the trait”) < 1. If the trait is at all useful in terms of determining guilt, it is sensible to suppose ψ_2_ > ψ_1_ and, under these circumstances, it is certainly reasonable to say there is evidence in favor of guilt as the probability of guilt has increased from a priori to a posteriori.

The question now is: does relative belief commit a prosecutor's fallacy? It might seem so as there will always be evidence of guilt when the trait is observed. Recall, however, that there are two parts to a relative belief inference whether estimation or hypothesis assessment, namely, we must also say something about the accuracy of the inference. Under these circumstances we have that ψ(“defendant has the trait”) = “guilty” but it is clear that C_Ψ , γ_(“defendant has the trait”) → {“guilty,” “not guilty”} as ϵ → 0 for any *γ* > 0. So for small ϵ the estimate has no accuracy at all! Furthermore, if we elected instead to assess the hypothesis *H*_0_: “guilty,” then the strength of this evidence is best assessed, since there are only two possible values, using the posterior probability *P* (“guilty” | “defendant has the trait”) and this converges to 0 as ϵ → 0 and again there is only very weak evidence in favor of guilt. So using the relative belief ratio to assess evidence, together with a measure of the strength of the evidence, protects against the prosecutor's fallacy as we will surely not convict based upon evidence in favor of guilt that is considered weak.

But the situation is more complicated than this yet and exposes a clear distinction between taking a decision-based approach and an evidential one. For consider the problem where ϵ corresponds to the proportion of individuals infected with a deadly infectious disease and *ψ*_1_ , *ψ*_2_ correspond to the probabilities of a test for infection being positive in the noninfected and infected populations, respectively. A good test will of course have *ψ*_2_ > *ψ*_1_ and so we are in exactly the same situation as, for a patient with a positive test, relative belief will record that there is evidence the patient is infected. Even if this is weak evidence, however, it would seem somewhat foolhardy to simply ignore the evidence.

A standard approach in this simple classification problem is to estimate *ψ* using the value that maximizes the posterior, called the MAP (maximum a posteriori) estimate. For ϵ small enough, this will declare the defendant innocent and the patient noninfected. In the former case this is reasonable but surely not in the latter case. It would seem that a categorical statement is not what is wanted from a statistical procedure in such problems. Undoubtedly decisions will be ultimately be made and these decisions may, for good reasons, ignore what the evidence says, but the additional criteria that come into play in making decisions are not statistical in nature. What is wanted from a theory of statistics is a statement concerning what the evidence indicates and, in addition, how strong that evidence is.

## 4 Conclusions

A broad outline of relative belief theory has been described here. The inferences have many nice properties like invariance under reparameterizations and a wide variety of optimal properties in the class of all Bayesian inferences. The papers Evans [11], Evans, Guttman, and Swartz, [15], Evans and Shakhatreh [22], Evans and Jang [18] and Baskurt and Evans [1] are primarily devoted to development of the theory. Many of these papers contain applications to specific problems but also see Evans, Gilula and Guttman [19], Cao, Evans and Guttman [5] and Muthukumarana and Evans [24]. Evans [13] presents a full development of relative belief theory together with procedures for model checking and checking for prior-data conflict.

It is worth emphasizing that for practitioners there are two ingredients that need to be specified to apply the theory of relative belief to statistical analyses, namely, the model {*f*_*θ*_ : *θ* ∈ Θ} and the prior *π*. Neither of these ingredients is necessarily determined by the application. In the end they are choices made by the practitioner which hopefully represent good judgment. In the event that these are poor choices, then it can be expected that the inferences may be erroneous and this is why the activities of model checking and checking for prior-data conflict are so important. If after these checks there is no reason to reject the choices made, then inference can proceed and relative belief gives an unambiguous approach to this. This lack of ambiguity is important as the failure of theories of inference to effectively solve inference problems leads to doubts as to the validity of inferences drawn on an ad hoc basis. The validity of relative belief inferences, once the basic principles are accepted, then rests with the choices made for the model and prior. Of course, it can never be said that these choices are “correct” only that they are not substantially wrong. These choices are essentially subjective in nature but the theory gives us tools for assessing any bias that the choices may have introduced into the analysis. This is the most we can expect from any theory of statistical inference.
